# Evaluation of the In2care Mosquito Station against *Culex quinquefasciatus* mosquitoes (Diptera: Culicidae) under semifield conditions

**DOI:** 10.1093/jme/tjae124

**Published:** 2024-10-07

**Authors:** Eva A Buckner, Ana L Romero-Weaver, Sierra M Schluep, Shawna K Bellamy, Rebecca A Zimler, Natalie L Kendziorski, Daviela Ramirez, Shelley A Whitehead

**Affiliations:** Florida Medical Entomology Laboratory, Department of Entomology and Nematology, Institute of Food and Agricultural Sciences, University of Florida, Vero Beach, FL, USA; Florida Medical Entomology Laboratory, Department of Entomology and Nematology, Institute of Food and Agricultural Sciences, University of Florida, Vero Beach, FL, USA; Florida Medical Entomology Laboratory, Department of Entomology and Nematology, Institute of Food and Agricultural Sciences, University of Florida, Vero Beach, FL, USA; Florida Medical Entomology Laboratory, Department of Entomology and Nematology, Institute of Food and Agricultural Sciences, University of Florida, Vero Beach, FL, USA; Florida Medical Entomology Laboratory, Department of Entomology and Nematology, Institute of Food and Agricultural Sciences, University of Florida, Vero Beach, FL, USA; Florida Medical Entomology Laboratory, Department of Entomology and Nematology, Institute of Food and Agricultural Sciences, University of Florida, Vero Beach, FL, USA; Florida Medical Entomology Laboratory, Department of Entomology and Nematology, Institute of Food and Agricultural Sciences, University of Florida, Vero Beach, FL, USA; Florida Medical Entomology Laboratory, Department of Entomology and Nematology, Institute of Food and Agricultural Sciences, University of Florida, Vero Beach, FL, USA

**Keywords:** *Culex quinquefasciatus*, West Nile virus, pyriproxyfen autodissemination, integrated mosquito management, In2Care Mosquito Station

## Abstract

*Culex quinquefasciatus* is an important mosquito vector responsible for the transmission of filarial worms, arthropod-borne viruses like Oropouche, St. Louis encephalitis, and West Nile and protozoans that cause avian malaria. Due to insecticide resistance documented in *Cx. quinquefasciatus* populations worldwide, integrated vector management programs can benefit from new strategies to control this species. The In2Care Mosquito Station (In2Care station), a commercially available dissemination station containing pyriproxyfen (PPF) and *Beauveria bassiana* spores, has been shown to be effective against skip-ovipositing *Aedes aegypti* and *Aedes albopictus* in previously conducted semifield and field trials. To determine the potential of *Cx. quinquefasciatus* adult females to autodisseminate PPF and if the In2Care station could be used for *Cx. quinquefasciatus* control, we assessed its efficacy in a semifield setting against wild *Cx. quinquefasciatus*. We found that the In2Care station was attractive to gravid *Cx. quinquefasciatus* females, with a significantly higher percentage of egg rafts laid in the In2Care station compared to alternative ovipots. Adult females successfully autodisseminated PPF from the In2Care station to surrounding ovipots, leading to a significant increase in mosquito emergence inhibition. Additionally, adult *Cx. quinquefasciatus* exposure to *B. bassiana* spores significantly reduced mosquito survivorship. These results suggest that the In2Care station may be effective against *Cx. quinquefasciatus* in addition to *Ae. aegypti* and *Ae. albopictus*. Additional field evaluations are needed to assess impacts at the population level.

## Introduction

*Culex quinquefasciatus* Say is a widespread mosquito species found throughout most of the tropical and subtropical regions of the world, which has been implicated in the transmission of numerous human, domestic animal, and wildlife pathogens, including those that cause St. Louis encephalitis disease, West Nile disease, Oropouche fever, lymphatic filariasis, and avian malaria (Hoch et al. 1987, Savage et al. 1993, Foster and Walker 2002, Godsey et al. 2005, LaPointe et al. 2005, McGregor et al. 2021). West Nile disease, in particular, is the leading mosquito-borne disease in the continental United States (CDC 2023), and *Cx. quinquefasciatus* is considered to be the primary West Nile virus (WNV) vector in suburban and urban areas in the southeastern United States (AMCA 2021). Within Florida, an average of 22 WNV human cases are reported annually (Day 2023), though sporadic outbreaks can lead to higher numbers of human cases. For example, a WNV outbreak in 2020, during which multiple WNV-positive *Cx. quinquefasciatus* pools were collected, resulted in 50 human cases primarily in suburban and urban areas in southern Florida (FDOH 2023).

Considering no vaccine is currently available for WNV in humans, controlling WNV mosquito vectors like *Cx. quinquefasciatus* is essential to preventing and reducing human disease incidence. However, *Cx. quinquefasciatus* control can prove difficult. Populations worldwide have been shown to be resistant to many insecticides used against adults (Chandre et al. 1998, Corbel et al. 2007, Sarkar et al. 2009, Low et al. 2013, Lopes et al. 2019). In the United States, *Cx. quinquefasciatus* has been shown to be resistant to multiple classes of insecticides and active ingredients used against adults (Liu et al. 2009, Richards et al. 2017, Burgess et al. 2022). In Florida, resistance to etofenprox, permethrin, pyrethrum, and sumithrin has been documented in *Cx. quinquefasciatus* populations (Liu et al. 2009, Richards et al. 2017, Lucas et al. 2020).

In addition to being resistant to adulticides, the propensity for *Cx. quinquefasciatus* to utilize container habitats for immature development can make them difficult to control. While not exclusively a container-inhabiting mosquito, numerous studies have documented the presence of *Cx. quinquefasciatus* mosquitoes in a variety of container habitats like tires, flowerpots, bird baths, and discarded items often co-occurring with exclusive container-inhabiting mosquito species like *Aedes aegypti* (Linnaeus) and *Aedes albopictus* (Skuse) (Yee 2008, Hribar and Whiteside 2011, Leisnham et al. 2014). These containers can be difficult to reach using traditional larviciding methods; therefore, an integrated mosquito management (IMM) that utilizes all available control methods against multiple mosquito life stages may be helpful for controlling *Cx. quinquefasciatus* mosquitoes (AMCA 2021).

Autodissemination of pyriproxyfen (PPF), a potent juvenile hormone analogue and larvicide, by adult *Ae. aegypti* and *Ae. albopictus* has been shown to reduce adult mosquito emergence (Estrada and Mulla 1986, Devine et al. 2009, Caputo et al. 2012, Ohba et al 2013). When applied at neighborhood (Abad-Franch et al. 2015, Paris et al. 2023) and city (Abad-Franch et al. 2017) levels, PPF autodissemination has been shown to reduce *Ae. aegypti* and *Ae. albopictus* mosquito populations, with the potential to block disease transmission (Abad-Franch et al. 2017). Additionally, recently Garcia et al. (2020) documented a significant reduction in adult *Ae. aegypti* and *Cx. quinquefasciatus* density due to a PPF autodissemination-based intervention in a 16-month-long study in Brazil, suggesting that PPF autodissemination may also be a tool to consider including in IMM plans against *Cx. quinquefasciatus*. However, it is unclear if the reduction in adult *Cx. quinquefasciatus* observed in Garcia et al. (2020) was due only to *Ae. aegypti* transferring PPF to larval habitats containing *Cx. quinquefasciatus* or if autodissemination of PPF by adult *Cx. quinquefasciatus* also contributed to the mosquito reduction.

Unlike container-inhabiting mosquitoes like *Ae. aegypti* and *Ae. albopictus*, gravid *Culex* female mosquitoes do not exhibit skip-oviposition and instead lay eggs from each gonotrophic cycle all at once in a raft on the water’s surface in an oviposition site (Clements 1992). The PPF autodissemination potential of *Culex* mosquitoes can, therefore, be expected to be lower compared to *Ae. aegypti* and *Ae. albopictus*. Nevertheless, *Cx. quinquefasciatus* gravid females have been documented to visit several potential locations before choosing a preferred oviposition site (Braks et al. 2007, Allgood and Yee 2017). If a gravid *Cx. quinquefasciatus* female visits a PPF autodissemination station during the search for an ideal oviposition site, then presumably any oviposition sites visited after the PPF autodissemination station would be contaminated with PPF by the female. In a laboratory setting, Mbare et al. (2014) demonstrated that gravid *Cx. quinquefasciatus* females can transfer lethal concentrations of PPF to oviposition sites with small volumes of water. The size of the larval habitat will determine if enough PPF has been transferred by the female to cause adult emergence inhibition. In lab and field studies, PPF has been shown to successfully inhibit adult *Cx. quinquefasciatus* emergence (Adames and Rovira 1993, Chavasse et al. 1995, Ali et al. 1999, Nayar et al. 2002). However, no semifield studies have evaluated the potential for *Cx. quinquefasciatus* adult females to autodisseminate PPF to oviposition sites and the subsequent impact on adult emergence.

Therefore, the purpose of our study was to investigate the potential of *Cx. quinquefasciatus* females to autodisseminate PPF to oviposition sites within a semifield setting and the resulting impact for using the In2Care Mosquito Station (In2Care station). The In2Care station combines the pupicidal and autodissemination ability of PPF with the adulticidal effects of the entomopathogenic fungus *Beauvaria bassiana* to kill both immature and adult mosquitoes. In field settings, a reduction of *Ae. aegypti*, *Ae. albopictus*, and *Ae. notoscriptus* mosquito populations using In2Care stations has been reported (Buckner et al. 2017, 2021, Khater et al. 2022, Paris et al. 2023).

We used the In2Care station in our evaluation because while designed as a control tool against skip-ovipositing, container-inhabiting *Aedes* mosquitoes, observations made during field trials suggest that they may also be attractive oviposition and resting sites for *Cx. quinquefasciatus* (Su et al. 2020, Buckner et al. 2021). *Culex quinquefasciatus* mosquitoes resting or ovipositing inside the In2Care station could result in PPF contamination and subsequent autodissemination. Therefore, mosquito release and recapture experiments were conducted to assess the combined impacts of PPF and *B. bassiana* on wild *Cx. quinquefasciatus* using methods like those previously used in Buckner et al. (2017). The results of this study demonstrate that gravid *Cx. quinquefasciatus* mosquitoes can successfully autodisseminate PPF within semifield settings, leading to a reduction in emergence of *Cx. quinquefasciatus* adults from oviposition containers. These results suggest that the In2Care station could potentially be used as part of IMM plan against *Cx. quinquefasciatus* mosquitoes in addition to *Ae. aegypti*, *Ae. albopictus*, and *Ae. notoscriptus*.

## Materials and Methods

### In2Care Mosquito Station

Experiments used the black plastic In2Care stations and In2Mix sachets (EPA Reg. No. 91720-1) containing 74.03% PPF and 10% *B. bassiana* strain GHA as active ingredients. Stations consisted of a black polypropylene UV-resistant container with a 5 liter volume capacity, a plastic floater, and a removable lid leaving a 5 cm opening to allow entry and exit of mosquitoes (https://www.in2care.org/how-to-use/). In2Care stations were treated with In2Mix one day before the onset of the experiment following the directions for use on the label. Each station was filled with 4.5 liters of clean tap water, the In2Mix-treated netting strip was taken out of the refill sachet and attached to the floater, and the floater was gently placed on the water surface. The remaining In2Mix powder and the two yeast tablets from the refill sachet were added to water in the station.

### Study Site

The study was conducted at the University of Florida, Institute of Food and Agricultural Sciences (UF/IFAS) Florida Medical Entomology Laboratory in Vero Beach, Florida. Experiments were conducted inside semifield enclosures from 18 June to 7 November 2019. The follow-up forced exposure assays were conducted from 22 September to 5 October 2020. Control and experiment replicates were set up inside the semifield system of 2.44 m^2^ tents screened with no-see-um netting exposed to ambient climate conditions. Temperature and humidity were recorded during experimental releases by a weather station < 1 km away (personal weather station KFLVEROB519 on www.wunderground.com).

### Mosquitoes

The *Cx. quinquefasciatus* mosquitoes used in experiments were collected from the field as eggs rafts from Bay, Pinellas, and Polk counties in Florida in the summer and autumn of 2019 and September 2020 and shipped overnight to UF/IFAS Florida Medical Entomology Laboratory inside sealed petri dishes lined with moist filter paper. Upon receipt, egg rafts were set in separate 40.6 × 15.4 × 6.4 cm enamel pans containing approximately 2 liters of water and larval diet of 1:1 by weight of lactalbumin and Brewer’s yeast within a walk-in insectary maintained at a temperature of 27 ± 2 °C and 50–60% relative humidity (RH). Larval development was monitored, and larval diet was added ad libitum. Once larvae were third or fourth instar, they were sight identified to species using the Darsie and Morris (2003) morphological key. Only the larvae identified as *Cx. quinquefasciatus* were utilized for this study. Pupae were collected daily and transferred to a water-filled cup inside a 30.5 × 30.5 × 30.5 cm adult rearing cage (BioQuip, Rancho Dominguez, CA). Once mosquitoes emerged as adults, they were provided with constant access to a cup containing a cotton ball soaked with a 10% sucrose solution.

Three- to five-days-old mosquitoes were provided a bloodmeal from a live chicken and were allowed to feed for 45 min. The University of Florida Institutional Animal Care and Use Committee (IACUC) reviewed and approved all procedures involving animals (IACUC Protocol # 201807682). Less than 2 h after blood-feeding, groups of approximately 50 visibly blood-fed female mosquitoes were collected using a mouth aspirator (John W. Hock Company, Gainesville, FL) and released into separate rearing cages that each contained a cotton ball soaked with a 10% sucrose solution. Three to four days after blood-feeding, gravid females were released into a 2.44 m^2^ tent screened with no-see-um netting exposed to ambient climate conditions, which contained either a control or treatment experimental setup described below.

### Experimental Setup

**Fig. 1.**
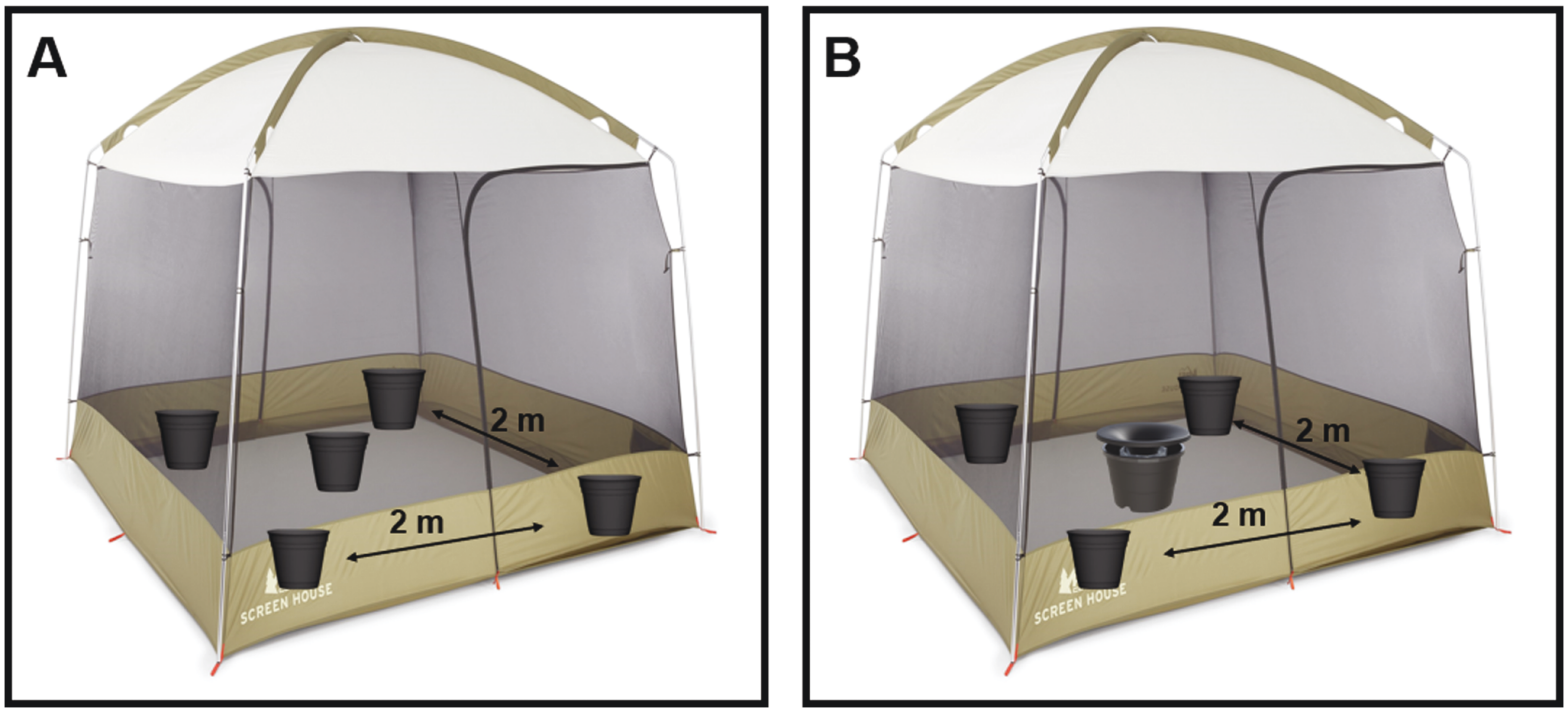
Semifield experimental setup in A) control replicates containing four ovipots placed in a 2 × 2 m square with one ovipot in the center and B) treatment replicates containing four ovipots placed in a 2 × 2 m square with one In2care Mosquito Station in the center of the four ovipots.

In the control experiment, four ovipots (black plastic flowerpots 15.2 cm in diameter, 13.9 cm depth) were placed inside the tent in a 2 × 2 m square. Another ovipot was placed at the center of the square (Fig. 1A). A clear glass bowl (15.2 cm diam, 7.6 cm depth) was placed inside each ovipot to hold water and to allow easy counting of egg rafts, pupae, and adults, as well as PPF decontamination after experiments. Four hundred milliliter of tap water, larval food (0.1 mg of 1:1 lactalbumin and Brewer’s yeast), and 20 second instar *Cx. quinquefasciatus* larvae were added to each glass bowl. Lastly, a cup containing a cotton ball soaked with a 10% sucrose solution was placed inside the tent to provide a sugar source for the free flying mosquitoes. The treatment experiment set-up was the same as described for the control experiment except the ovipot at the center of the four ovipots was replaced with an In2Care station filled with 4.5 liters of water and treated with In2Mix as described above. Twenty second instar *Cx. quinquefasciatus* larvae were then added to the In2Care station at the center of the treatment experiment set-up (Fig. 1B).

### Release–Recapture Procedures

Control and treatment experiments were repeated five times using groups of approximately 50 gravid *Cx. quinquefasciatus* mosquitoes. Experimental release dates were influenced by the availability of 50 *Cx. quinquefasciatus* gravid mosquitoes. Control experiments were conducted from 18 June to 18 September 2019, prior to treatment experiments to reduce the likelihood of PPF and *B. bassiana* contamination of the tents. The treatment experiment releases took place from 23 September to 18 October 2019. All releases took place in the morning between 9 and 11 AM. Approximately 48 h later, adult mosquitoes were recaptured using a handheld, motorized aspirator (John W. Hock Company) and placed in 8-ounce paper cup containers (Webstaurantstore, Lancaster, PA) covered with netting. A cotton ball soaked with 10% sucrose solution was placed on top of each container, and the daily adult mosquito survivorship was recorded for 21 days. The control experiment replicates were monitored for daily survivorship from 20 June to 13 October 2019. The treatment experiment replicates were monitored for daily survivorship from 25 September to 7 November 2019.

Once adult aspiration was completed, the ovipot bowls and In2Care stations containing larvae were removed from the tent. The number of rafts laid in each bowl and In2Care station was recorded. All glass bowls were covered with a nylon stocking to capture any emerging mosquitoes while monitoring post-experiment mosquito development. Bowls were checked daily until all larvae in each bowl either pupated and emerged or died. The number of dead larvae and pupae and emerged adults from each bowl was recorded.

### Impact Measurements

Station attractiveness in each treatment experiment replicate was examined by using *Culex* oviposition as a proxy for adult visitation. The percentage of rafts laid inside the In2Care station was compared to the percentage of rafts laid in the ovipots of each treatment experiment replicate. It was assumed that equal raft distribution across all water filled containers indicated equivalent attractiveness to egg-laying mosquitoes. Adult mosquito emergence rates (% *Culex* mosquitoes developed from the 20 added larvae) were used as a proxy for the larvicidal impact of the PPF in the In2Mix. Pyriproxifen autodissemination was determined by comparing the mean percentage of adult emergence between the ovipots placed around the In2Care station in the treatment experiment replicates and the ovipots in the control experiment replicates. The effect of *B. bassiana* spores on adult survivorship was determined by scoring the daily survivorship of retrieved adult cohorts from all replicates for 21 days post-exposure and comparing the mean cumulative daily survival rates for each experimental group.

There was variability and an inadequate number of mosquitoes recaptured in the treatment replicates to enable the detection of any impact of *B. bassiana* spores on adult mosquito survivorship compared to the control replicates. Therefore, extra mosquito bioassays were conducted in September 2020 to assess the impact of the *B. bassiana* spores on adult *Cx. quinquefasciatus* mosquito survival. Standard World Health Organization (WHO) susceptibility bioassay tubes (125 mm length, 44 mm diameter, 16-mesh gauze at either end) were lined on the inside with In2Mix-treated netting stapled to filter paper (WHO 2016). Clean WHO tubes lined with clean filter paper were used for control replicates to score baseline mosquito survival. Cohorts of 25 *Cx. quinquefasciatus* adult females (3–6 days old) collected as egg rafts on-site at UF/IFAS Florida Medical Entomology Lab and reared to adulthood using the methods described above were added to each tube and exposed for 3 min, according to WHO protocol. Each cohort was subsequently transferred to 8-ounce paper cup containers covered with netting. A cotton ball soaked with 10% sucrose solution was placed on top of the netting. The containers of mosquitoes were then housed in a screen room, which allowed exposure to ambient climate conditions. Temperature and humidity were recorded using the same weather station used for experiments in the year before. Survivorship was scored daily for each group for 14 days, after which all exposed test replicate individuals had succumbed to fungal infection and died. In total, five control replicates with clean tubes and five test replicates with In2Mix-treated netting were conducted. The effect of the *B. bassiana* spores on *Cx. quinquefasciatus* survivorship was determined by comparing the mean cumulative daily survivorship between the control and treatment groups.

### Statistical Analysis

Statistical analysis was performed similarly to Buckner et al. (2017) using JMP 11.2.0 software (SAS Institute Inc., Cary, NC) and SPSS software (IBM SPSS Statistics 22.0). The normality of daily mean temperature, raft counts, and adult emergence data was analyzed using Shapiro–Wilk tests. Homogeneity of variances was tested using Levene’s tests. For non-normal data with unequal variances, Kruskal–Wallis (Mann–Whitney U) tests were used to investigate significant differences between control and treatment experiments. The mean percentage of egg rafts laid inside the In2Care station was compared to the mean percentage of rafts laid in each ovipot for the five treatment replicates using a Mann–Whitney U test. The impact of PPF autodissemination in the treatment experiment was determined by comparing the mean percentage of adult emergence inhibition from the ovipots surrounding the In2Care station in the five treatment experiment replicates to the mean percentage of adult emergence inhibition from the ovipots in the five control experiment replicates using a Mann–Whitney U test.

For adulticidal impact assessments, the number of recaptured mosquitoes was recorded relative to the number of mosquitoes released. Survivorship data were entered per individual recaptured mosquito, noting the observed time point (d) of death for 21 days. Kaplan–Meier survival analyses were used to compute survival functions from the nonparametric life-time data. To test the null hypothesis that survival functions did not differ across treatment groups, Kaplan–Meier pairwise analysis was utilized with the log-rank test in SPSS. Since these experiments were not paired replicates, as they were not initiated on the same day or using the same mosquito rearing cohorts, survival curves were compared for the pooled replicates of control and treatment experiments. Kaplan–Meier test statistic results were compared with a chi squared distribution with one degree of freedom to yield a *P* value.

## Results

The control semifield experiments were conducted from 18 June 2019 to 18 September 2019, and the treatment semifield experiments were conducted from 23 September 2019 to 18 October 2019. The daily mean temperature ± SE during the control experiments (27.59 ± 0.10 °C) was significantly higher than the daily mean temperature during the treatment experiments (26.26 ± 0.19 °C; *x*^2^ = 25.50, *P* = < 0.0001). The lowest recorded nighttime temperature during the 2-day experiment releases was 11.28 °C in November, and the highest recorded daytime temperature was 35.56 °C in June. The daily mean RH ± SE during the control experiments (45.87 ± 1.58%) was significantly lower than the daily RH during the treatment experiments (68.31 ± 1.69%; *x*^2^ = 35.76, *P* = < 0.0001). The lowest recorded RH during the experiments was 1% in June, and the highest recorded RH was 84% in November. The temperature and RH recorded during the experiments show that the experiments were conducted under natural and varying ambient climate conditions.

### In2Care Station Attraction

**Fig. 2.**
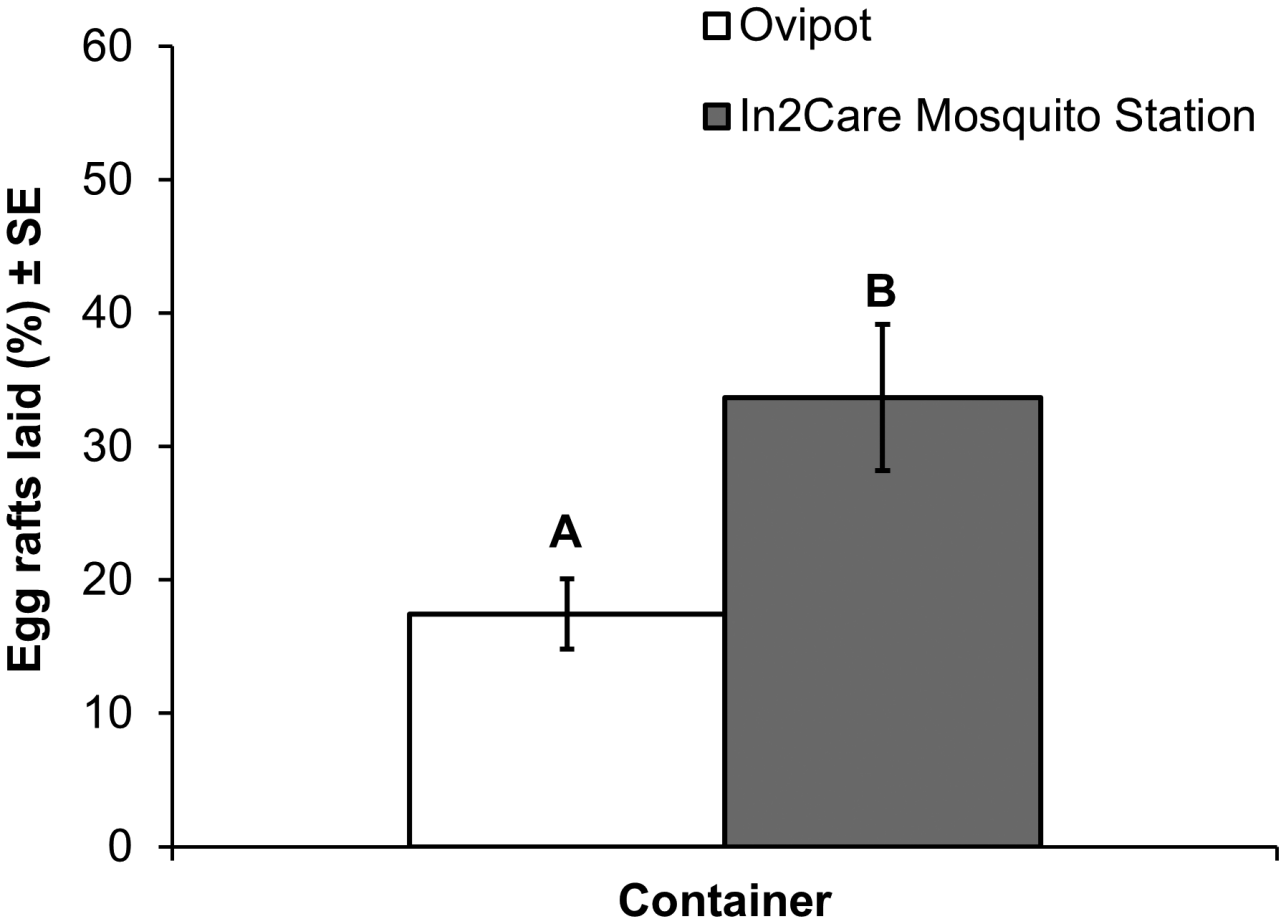
Mean percentage of *Culex quinquefasciatus* egg rafts laid (±SE) in ovipot versus In2Care Mosquito Station after 2-day release in the screenhouse for treatment replicates. Different letters above bars indicate significant differences (*P* = 0.016).

In the treatment experiment replicates, gravid *Cx. quinquefasciatus* females laid an average of four egg rafts in ovipots and an average of seven egg rafts in the In2Care stations. Statistical analyses showed that the mean percentage of egg rafts laid by *Cx. quinquefasciatus* in In2Care stations (34 ± 5%) was significantly higher than the mean percentage laid in ovipots (18 ± 3%) (*P* = 0.016; Fig. 2). This indicates that the In2Care stations were more attractive to *Cx. quinquefasciatus* than the alternative oviposition site provided in the treatment experiment and confirms that the presence of the PPF- and *B. bassiana*-treated floater in the stations does not negatively affect *Cx. quinquefasciatus* oviposition attraction.

### Larvicidal Impacts

In the control replicates, on average 87.3 ± 1.8% of the added larvae fully developed and emerged as adult mosquitoes. In contrast, in the In2Mix-treated In2Care stations, 0% adult emergence was observed for all replicates. All *Cx. quinquefasciatus* larvae were killed in the late L_4_ stage or pupal stage, and dead pupae showed a distinct black color and de-curling of the tail, which is distinct for PPF-induced molting inhibition. The observed 100% adult emergence inhibition confirms the larvicidal impact of the PPF on wild *Cx. quinquefasciatus*.

### Autodissemination Impacts

**Fig. 3.**
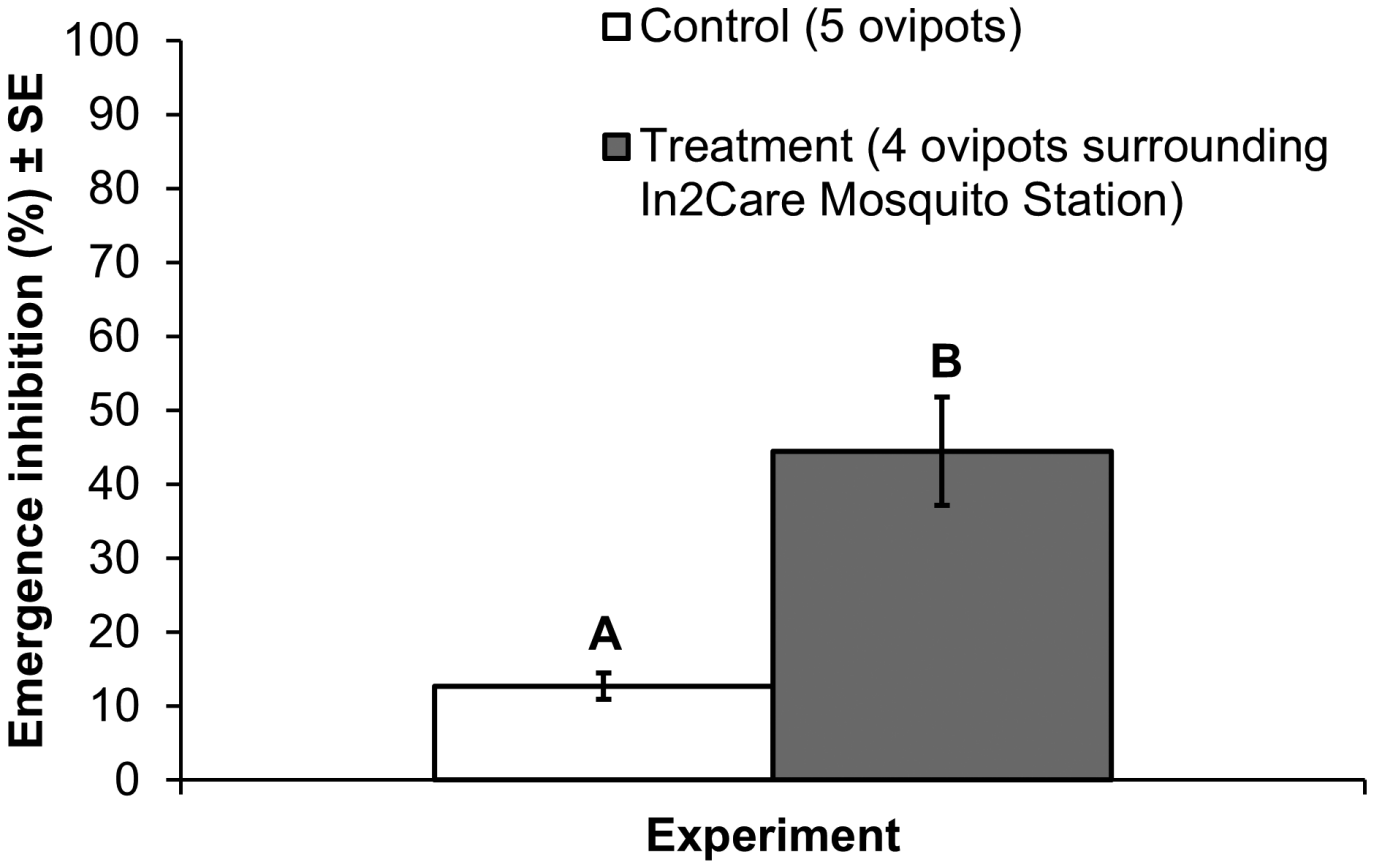
Mean (±SE) adult emergence inhibition rates of *Culex quinquefasciatus* larvae (*n* = 20) added to five ovipots in control replicates versus four ovipots surrounding an In2Care Mosquito Station in treatment replicates. Different letters above bars indicate significant differences (*P *< 0.001).

On average, the emergence inhibition rate was 45 ± 7% from the four ovipots placed around the In2Care station in treatment experiment replicates, which was significantly higher than the emergence inhibition rate of 13 ± 2% from the five ovipots in the control experiment replicates (*P* < 0.001; Fig. 3). In other words, 55% of *Cx. quinquefasciatus* larvae in the ovipots surrounding the In2Care station died due to PPF autodissemination. The PPF-specific pupicidal effect observed in treatment experiment replicates, black uncurled dead pupae, confirmed that PPF was successfully spread from the station to the surrounding ovipots.

### Adulticidal Impacts

**Fig. 4.**
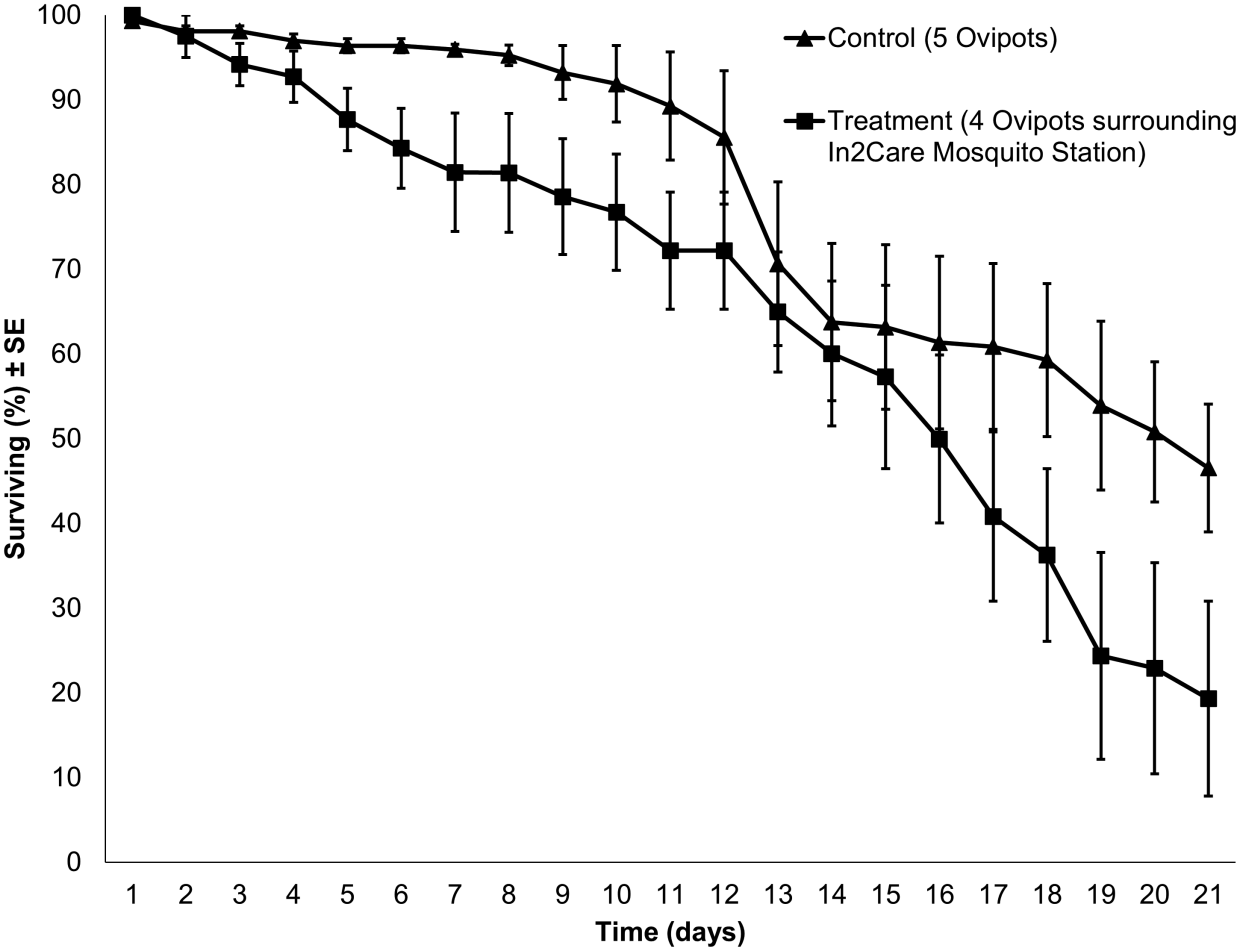
Mean (±SE) cumulative % survival of recaptured adult *Culex quinquefasciatus* cohorts released inside tents with five ovipots or one In2Care Mosquito Station and four ovipots, which did not significantly differ (*P* = 0.131).

**Fig. 5.**
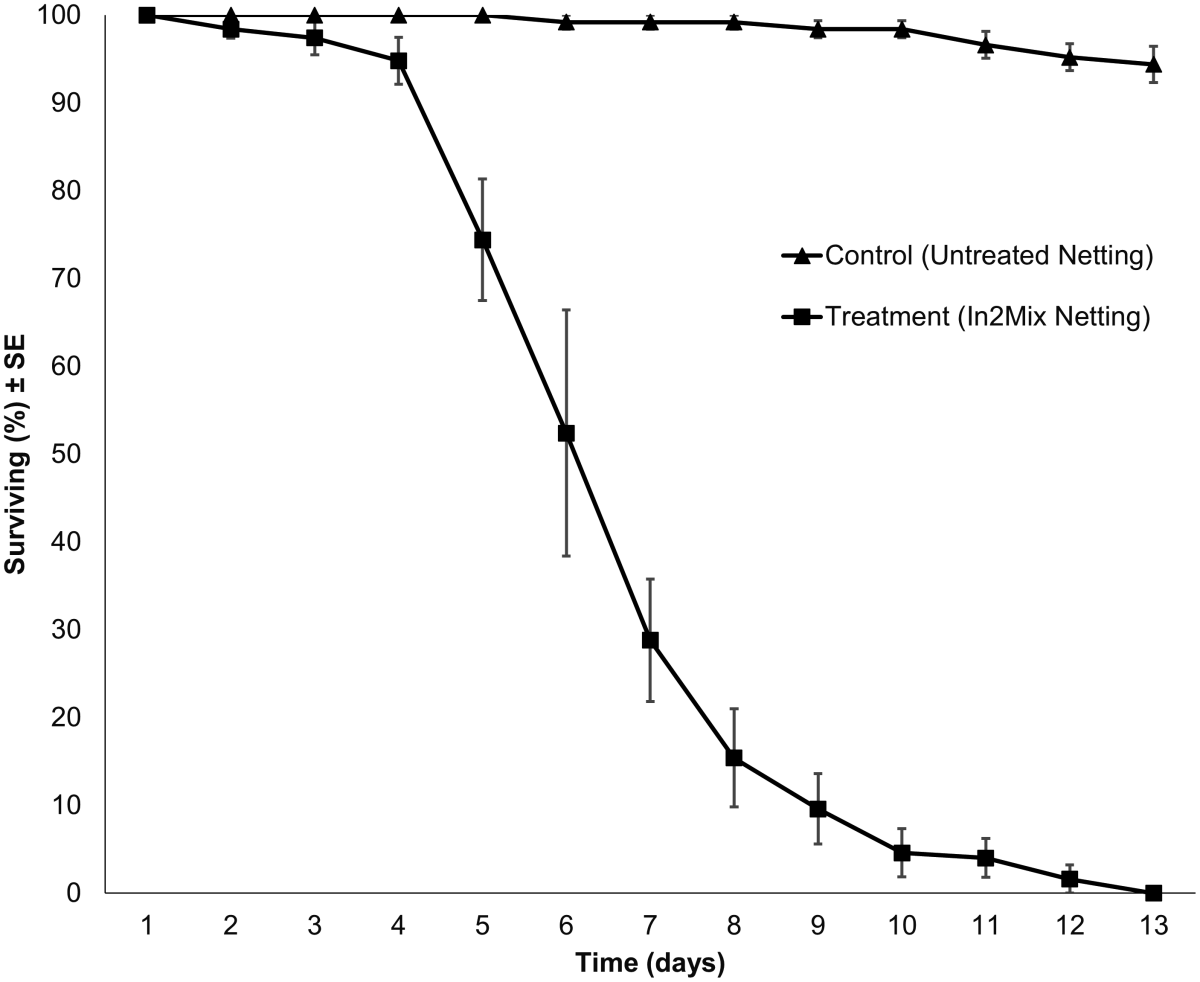
Mean (±SE) cumulative % survival of adult *Culex quinquefasciatus* cohorts (*N* = 25, *n* = 5) after 3 min forced exposure to clean netting or In2Mix-treated netting used in In2Care Mosquito Stations in WHO bioassay tubes, which significantly differed (*P* < 0.001).

We found no significant difference in adult survivorship between the semifield experimental groups (*P* = 0.131; Fig. 4), which was most likely influenced by the low mean adult recapture rate in treatment replicates (30 ± 11%) compared to control replicates (88 ± 14%). Follow-up bioassay results showed that *Cx. quinquefasciatus* mosquito survivorship was significantly reduced after exposure to In2Mix-treated netting containing *B. bassiana* spores (Fig. 5). The survival curves of In2Mix-exposed cohorts showed a sigmoid decline that is typical of *Beauveria*-induced lethality, killing the majority of mosquitoes within eight days, and all *Cx. quinquefasciatus* females within 13 days. The mean control survival was still 94% on average 13 days post-exposure. Kaplan–Meier analyses confirmed that the observed differences between the survival curves of the control and treatment cohorts were highly significant (*P* < 0.001). Mean temperature was 25.44 °C and the mean RH was 84.5% during bioassay monitoring. These findings confirm the adulticidal efficacy of In2Mix-baited In2Care stations for *Cx. quinquefasciatus* mosquitoes under natural ambient climate conditions.

## Discussion

We documented a significantly higher mean percentage of *Cx. quinquefasciatus* egg rafts laid in an In2Care station compared to alternative ovipots in our semifield experiments, which demonstrates that the In2Care station is an attractive oviposition site for gravid *Cx. quinquefasciatus* adult females. The consistent larvicidal impact observed indicates that In2Care stations can act as an egg sink and prevent adult emergence from egg rafts deposited by visiting *Cx. quinquefasciatus* females. We also documented a significantly higher mean percentage of adult emergence inhibition for ovipots in treatment experiments compared to ovipots in control experiments, which shows that adult *Cx. quinquefasciatus* females successfully autodisseminated PPF from In2Care station to surrounding ovipots. Lastly, while we did not see a significant difference in mortality rates between control and treatment recaptured adults from our semifield experiments, we found that adult *Cx. quinquefasciatus* exposure to *B. bassiana* during forced bioassays significantly reduced survivorship. The fungus spores in the In2Mix-induced high mortality rates (>85%) in adult *Cx. quinquefasciatus* mosquitoes within 8 days post-exposure under ambient climate conditions.

Mosquito recapture rates in the semifield treatment replicates were lower than the control replicates likely leading to our inability to detect a significant impact of *B. bassiana* on adult survivorship in the treatment experiment. The low treatment recapture rate may have been influenced by ambient temperatures during the treatment replicates. Due to a limited number of tents available for the semifield experiments, treatment replicates were performed after control replicates with the same tents rather than the control and treatment experiments being performed concurrently. Also, issues with obtaining enough gravid *Cx. quinquefasciatus* mosquitoes to perform all needed control experiment replicates delayed conducting the treatment experiment further. As a result, a significantly lower mean daily temperature (26.26 °C) was recorded during the treatment replicates performed later in the year compared to the control replicates (27.59 °C). Mean daily RH was significantly higher during the treatment replicates (68.31%) compared to control replicates (45.87%). *Beauveria bassiana* fungal growth and virulence against mosquitoes has been shown to be correlated with cooler temperatures and higher RH rates (Hart and MacLeod 1955, Clark et al. 1968, Blanford et al. 2012, Popko et al. 2018). Possibly, we failed to recapture fungus-infected mosquitoes already experiencing the negative impacts of *B. bassiana*, and the mosquitoes collected were uninfected. Relatively higher survival rates of uninfected individuals could have diluted the impact of *B. bassiana* on the survivorship of treatment mosquitoes.

Additional limitations of our study include that it did not provide direct evidence of PPF transport by *Cx. quinquefasciatus* using a detection technique such as LC–MS/MS. We assumed PPF transfer based on adult emergence inhibition and the PPF-specific pupicidal effect, black uncurled dead pupae, observed in treatment experiment replicates. Also, experiments were conducted in a relatively small enclosure, and the attraction of the In2Care station was only compared to ovipots containing 0.1 mg of 1:1 lactalbumin and Brewer’s yeast. Gravid *Cx. quinquefasciatus* mosquitoes are known to rely heavily on olfactory clues for oviposition site selection. Water with high concentrations of organic matter acts as an oviposition attractant for this species (McCall and Eaton 2001). Future development on the targeted use of In2Care stations for *Culex* mosquito control should therefore include further testing of *Culex*-specific organic odor lures such as hay or additional microbial infusions. Field studies will be required to compare the attraction of In2Care stations to competitive oviposition sources found in real settings.

Our results show the PPF autodissemination potential of *Cx. quinquefasciatus* females within a semifield setting. However, adult emergence inhibition in the ovipots surrounding In2Care stations was less pronounced in this study performed with *Cx. quinquefasciatus* compared to previous studies performed with *Ae. aegypti* and *Ae. albopictus* (Buckner et al. 2017), which was probably caused by the lack of skip-oviposition behavior by *Cx. quinquefasciatus* (Clements 1992). Since *Cx. quinquefasciatus* gravid females have been documented visiting several potential locations before choosing a preferred oviposition site (Braks et al. 2007, Allgood and Yee 2017), the observed mosquito-driven dispersal of PPF may have been caused by gravid *Cx. quinquefasciatus* mosquitoes first visiting the In2Care station and subsequently laying eggs in the ovipots. Even within a short exposure period of two days, there was sufficient In2Care station mosquito visitation and PPF autodissemination to result in a statistically significant reduction in *Cx. quinquefasciatus* mosquitoes emerging from surrounding ovipots in the treatment experimental replicates compared to the negative control experimental replicates.

The high dispersal capacity of *Cx. quinquefasciatus*, which is in the range of kilometers (Medeiros et al. 2017), may require the need of large-scale deployment of PPF autodissemination stations at high densities to be effective in the field. For example, McNamara et al. (2024) recently conducted a field evaluation of In2Care stations deployed at a low density of three stations per acre against *Ae. aegypti* and *Cx. quinquefasciatus* in a north-central Florida neighborhood. No significant difference between the *Ae. aegypti* and *Cx. quinquefasciatus* population levels in the treatment and control areas was observed suggesting that the low-density In2Care station deployment was insufficient to reduce *Ae. aegypti* and *Cx. quinquefasciatus* density in the treatment area (McNamara et al. 2024).

Conversely, if PPF stations are deployed at high densities and in areas where container-inhabiting *Aedes* mosquitoes and *Cx. quinquefasciatus* co-occur, it may be possible that PPF autodissemination by container-inhabiting *Aedes* will increase the potential impact on *Cx. quinquefasciatus* in the field. *Culex quinquefasciatus*, *Ae. albopictus*, and/or *Ae. aegypti* often have overlapping distributions in suburban and urban areas and utilize the same containers for immature development (Yee 2008, Hribar and Whiteside 2011, Leisnham et al. 2014, Barrera et al. 2008). Pyriproxifen autodissemination by container-inhabiting *Aedes* to containers with both *Aedes* and *Cx. quinquefasciatus* larvae could reduce *Aedes* and *Cx. quinquefasciatus* adult emergence. For example, while unclear if due to PPF autodissemination by *Aedes* alone or both *Culex* and *Aedes*, Garcia et al. (2020) documented 56% reduction in *Cx. quinquefasciatus* adult density and 60% reduction in *Ae. aegypti* adult density in their 16-month, two-arm, cluster-randomized controlled trial of autodisseminated PPF in central-western Brazil. Additional field evaluations are needed to assess the impact of PPF autodissemination on *Cx. quinquefasciatus* mosquito populations in settings with and without co-inhabiting *Aedes* populations.

Since *Cx. quinquefasciatus* mosquitoes are vectors of disease-causing pathogens and difficult to control due to their potential cryptic container-inhabiting behavior and increasing levels of insecticide resistance, there is a need for novel control tools that can be used in IMM strategies. Our findings that In2Care stations can effectively lure and kill *Cx. quinquefasciatus* females and kill their offspring indicate that PPF autodissemination products like the In2Care station can be used as part of IMM strategies for *Cx. quinquefasciatus* mosquito control. Large scale field evaluations with varying densities of In2Care stations remain needed to assess the impact of the In2Care station on wild *Cx. quinquefasciatus* populations.

## References

[CIT0001] Abad-Franch F , Zamora-Perea E, Ferraz G, et al. 2015. Mosquito-disseminated pyriproxyfen yields high breeding-site coverage and boosts juvenile mosquito mortality at the neighborhood scale. PLoS Negl. Trop. Dis. 9(4):e0003702. https://doi.org/10.1371/journal.pntd.0003702 25849040 PMC4388722

[CIT0002] Abad-Franch F , Zamora-Perea E, Luz SL. 2017. Mosquito-disseminated insecticide for citywide vector control and its potential to block arbovirus epidemics: entomological observations and modeling results from Amazonian Brazil. PLoS Med. 14(1):e1002213. https://doi.org/10.1371/journal.pmed.1002213 28095414 PMC5240929

[CIT0003] Adames E , Rovira J. 1993. Evaluation of the juvenile growth regulator pyriproxyfen (S-31183) against three species of mosquitoes in Panama. J. Am. Mosq. Control Assoc. 9:452–453.

[CIT0004] Ali A , Chowdhury MA, Hossain MI, et al. 1999. Laboratory evaluation of selected larvicides and insect growth regulators against field-collected *Culex quinquefasciatus* larvae from urban Dhaka, Bangladesh. J. Am. Mosq. Control Assoc. 15(1):43–47. 10342267

[CIT0005] Allgood DW , Yee DA. 2017. Oviposition preference and offspring performance in container breeding mosquitoes: evaluating the effects of organic compounds and laboratory colonisation. Ecol. Entomol. 42(4):506–516. https://doi.org/10.1111/een.12412 28989226 PMC5625354

[CIT0006] American Mosquito Control Association (AMCA). 2021. Best practices for integrated mosquito management. Sacramento (CA): American Mosquito Control Association. https://www.mosquito.org/assets/pdf/hr_november_2021_amca_bmp_ma/.

[CIT0007] Barrera R , Amador M, Diaz A, et al. 2008. Unusual productivity of *Aedes aegypti* in septic tanks and its implications for dengue control. Med. Vet. Entomol. 22(1):62–69. https://doi.org/10.1111/j.1365-2915.2008.00720.x 18380655

[CIT0008] Blanford S , Jenkins NE, Christian R, et al. 2012. Storage and persistence of a candidate fungal biopesticide for use against adult malaria vectors. Malar. J. 11:354. https://doi.org/10.1186/1475-2875-11-354 23098323 PMC3506477

[CIT0009] Braks MAH , Leal WS, Cardé RT. 2007. Oviposition responses of gravid female *Culex quinquefasciatus* to egg rafts and low doses of oviposition pheromone under semi-field conditions. J. Chem. Ecol. 33(3):567–578. https://doi.org/10.1007/s10886-006-9223-8 17252215

[CIT0010] Buckner EA , Williams KF, Marsicano AL, et al. 2017. Evaluating the vector control potential of the In2Care^®^ Mosquito Trap against *Aedes aegypti* and *Aedes albopictus* under semifield conditions in Manatee County, Florida. J. Am. Mosq. Control Assoc. 33(3):193–199. https://doi.org/10.2987/17-6642R.1 28854105

[CIT0011] Buckner EA , Williams KF, Ramirez S, et al. 2021. A field efficacy evaluation of In2Care Mosquito Traps in comparison with routine integrated vector management at reducing *Aedes aegypti*. J. Am. Mosq. Control Assoc. 37(4):242–249. https://doi.org/10.2987/21-7038 34817613

[CIT0012] Burgess ER 4th , Lopez K, Irwin P, et al. 2022. Assessing pyrethroid resistance status in the *Culex pipiens* complex (Diptera: Culicidae) from the northwest suburbs of Chicago, Illinois using Cox regression of bottle bioassays and other detection tools. PLoS One 17(6):e0268205. https://doi.org/10.1371/journal.pone.0268205 35767519 PMC9242439

[CIT0013] Caputo B , Ienco A, Cianci D, et al. 2012. The “auto-dissemination” approach: a novel concept to fight *Aedes albopictus* in urban areas. PLoS Negl. Trop. Dis. 6(8):e1793. https://doi.org/10.1371/journal.pntd.0001793 22953015 PMC3429402

[CIT0014] (CDC) Centers for Disease Control and Prevention. 2023. About West Nile Virus. [accessed 2023 Sep 8]. https://www.cdc.gov/west-nile-virus/about/index.html.

[CIT0015] Chandre F , Darriet F, Darder M, et al. 1998. Pyrethroid resistance in *Culex quinquefasciatus* from West Africa. Med. Vet. Entomol. 12(4):359–366. https://doi.org/10.1046/j.1365-2915.1998.00120.x 9824819

[CIT0016] Chavasse DC , Lines JD, Ichimori K, et al. 1995. Mosquito control in Dar es Salaam. II. Impact of expanded polystyrene beads and pyriproxyfen treatment of breeding sites on *Culex quinquefasciatus* densities. Med. Vet. Entomol. 9(2):147–154. https://doi.org/10.1111/j.1365-2915.1995.tb00171.x 7787222

[CIT0017] Clark TB , Kellen WR, Fukuda T, et al. 1968. Field and laboratory studies on the pathogenicity of the fungus *Beauveria bassiana* to three genera of mosquitoes. J. Invertebr. Pathol. 11(1):1–7. https://doi.org/10.1016/0022-2011(68)90047-5 5654770

[CIT0018] Clements AN. 1992. The biology of mosquitoes. London: Chapman & Hall.

[CIT0019] Corbel V , N’Guessan R, Brengues C, et al. 2007. Multiple insecticide resistance mechanisms in *Anopheles gambiae* and *Culex quinquefasciatus* from Benin, West Africa. Acta Trop. 101(3):207–216. https://doi.org/10.1016/j.actatropica.2007.01.005 17359927

[CIT0020] Darsie RF Jr , Morris CD. 2003. Keys to the adult females and fourth instar larvae of the mosquitoes of Florida (Diptera, Culicidae). Fort Myers, FL: Florida Mosquito Control Association.

[CIT0021] Day JF. 2023. Florida arbovirus disease activity update. St. Charles, IL: Clarke; [accessed 2023 Oct 10]. https://www.clarke.com/florida-arboviral-disease-activity-report/.

[CIT0022] Devine GJ , Perea EZ, Killeen GF, et al. 2009. Using adult mosquitoes to transfer insecticides to *Aedes aegypti* larval habitats. Proc. Natl. Acad. Sci. U.S.A. 106(28):11530–11534. https://doi.org/10.1073/pnas.0901369106 19561295 PMC2702255

[CIT0023] Estrada JG , Mulla MS. 1986. Evaluation of two new insect growth regulators against mosquitoes in the laboratory. J. Am. Mosq. Control Assoc. 2(1):57–60. 2906962

[CIT0024] (FDOH) Florida Department of Health. 2023. Mosquito-borne disease surveillance. [accessed 2023 Aug 12]. https://www.floridahealth.gov/diseases-and-conditions/mosquito-borne-diseases/surveillance.html.

[CIT0025] Foster WA , Walker ED. 2002. Mosquitoes (Culicidae). In: Mullen G, Durden L, editors. Medical and veterinary entomology. New York (NY): Academic Press; p. 245–249.

[CIT0026] Garcia KKS , Versiani HS, Araújo TO, et al. 2020. Measuring mosquito control: adult-mosquito catches vs egg-trap data as endpoints of a cluster-randomized controlled trial of mosquito-disseminated pyriproxyfen. Parasit Vectors. 13(1):352. https://doi.org/10.1186/s13071-020-04221-z 32665032 PMC7362459

[CIT0027] Godsey MS Jr , Blackmore MS, Panella NA, et al. 2005. West Nile virus epizootiology in the southeastern United States, 2001. Vector Borne Zoonotic Dis. (Larchmont, N.Y.) 5(1):82–89. https://doi.org/10.1089/vbz.2005.5.82 15815153

[CIT0028] Hart MP , MacLeod DM. 1955. An apparatus for determining the effects of temperature and humidity on the germination of fungus spores. Can. J. Bot. 33(4):289–292.

[CIT0029] Hoch AL , Pinheiro FD, Roberts DR, et al. 1987. Laboratory transmission of Oropouche virus by *Culex quinquefasciatus* Say. Bull. Pan Am. Health Organ. 21(1):55–61. 3607353

[CIT0030] Hribar LJ , Whiteside ME. 2011. Productivity of container habitats for pupal *Culex quinquefasciatus* Say and *Culex nigripalpus* Theobald (Diptera: Culicidae) in the Florida Keys. Trends Entomol. 7:33–36.

[CIT0031] Khater EIM , Autry D, Gaines MK, et al. 2022. Field evaluation of autocidal gravid ovitraps and In2Care traps against *Aedes* mosquitoes in Saint Augustine, northeastern Florida. J. Fla. Mosq. Control Assoc. 69:48–54. https://doi.org/10.32473/jfmca.v69i1.130626

[CIT0032] LaPointe DA , Goff ML, Atkinson CT. 2005. Comparative susceptibility of introduced forest-dwelling mosquitoes in Hawai’i to avian malaria *Plasmodium relictum*. J. Parasitol. 91(4):843–849. https://doi.org/10.1645/GE-3431.1 17089752

[CIT0033] Leisnham PT , LaDeau SL, Juliano SA. 2014. Spatial and temporal habitat segregation of mosquitoes in urban Florida. PLoS One 9(3):e91655. https://doi.org/10.1371/journal.pone.0091655 24621592 PMC3951416

[CIT0034] Liu N , Xu Q, Li T, et al. 2009. Permethrin resistance and target site insensitivity in the mosquito *Culex quinquefasciatus* in Alabama. J. Med. Entomol. 46(6):1424–1429. https://doi.org/10.1603/033.046.0625 19960691

[CIT0035] Lopes RP , Lima JBP, Martins AJ. 2019. Insecticide resistance in *Culex quinquefasciatus* Say, 1823 in Brazil: a review. Parasit. Vectors 12(1):591. https://doi.org/10.1186/s13071-019-3850-8 31852489 PMC6921570

[CIT0036] Low VL , Chen CD, Lee HL, et al. 2013. Enzymatic characterization of insecticide resistance mechanisms in field populations of Malaysian *Culex quinquefasciatus* Say (Diptera: Culicidae). PLoS One 8(11):e79928. https://doi.org/10.1371/journal.pone.0079928 24278220 PMC3836847

[CIT0037] Lucas KJ , Bales RB, McCoy K, et al. 2020. Oxidase, esterase, and KDR-associated pyrethroid resistance in *Culex quinquefasciatus* field collections of Collier County, Florida. J. Am. Mosq. Control Assoc. 36(1):22–32. https://doi.org/10.2987/19-6850.1 32497474

[CIT0038] Mbare O , Lindsay SW, Fillinger U. 2014. Pyriproxyfen for mosquito control: female sterilization or horizontal transfer to oviposition substrates in *Anopheles gambiae* sensu stricto and *Culex quinquefasciatus*. Parasit. Vectors 7:280. https://doi.org/10.1186/1756-3305-7-280 24954695 PMC4082483

[CIT0039] McCall PJ , Eaton G. 2001. Olfactory memory in the mosquito *Culex quinquefasciatus*. Med. Vet. Entomol. 15(2):197–203. https://doi.org/10.1046/j.0269-283x.2001.00304.x 11434554

[CIT0040] McGregor BL , Connelly CR, Kenney JL. 2021. Infection, dissemination, and transmission potential of North American *Culex quinquefasciatus*, *Culex tarsalis*, and *Culicoides sonorensis* for Oropouche Virus. Viruses 13(2):226. https://doi.org/10.3390/v13020226 33540546 PMC7912852

[CIT0041] McNamara TD , Vargas N, McDuffie D, et al. 2024. Evaluation of the In2Care Mosquito Station at low deployment density: a field study to manage *Aedes aegypti* and *Culex quinquefasciatus* (Diptera: Culicidae) in North Central Florida. J. Med. Entomol 61(5):1190–1202. https://doi.org/10.1093/jme/tjae089 39093689

[CIT0042] Medeiros MC , Boothe EC, Roark EB, et al. 2017. Dispersal of male and female *Culex quinquefasciatus* and *Aedes albopictus* mosquitoes using stable isotope enrichment. PLoS Negl. Trop. Dis. 11(1):e0005347. https://doi.org/10.1371/journal.pntd.0005347 28135281 PMC5300284

[CIT0043] Nayar JK , Ali A, Zaim M. 2002. Effectiveness and residual activity comparison of granular formulations of insect growth regulators pyriproxyfen and s-methoprene against Florida mosquitoes in laboratory and outdoor conditions. J. Am. Mosq. Control Assoc. 18(3):196–201. 12322941

[CIT0044] Ohba SY , Ohashi K, Pujiyati E, et al. 2013. The effect of pyriproxyfen as a “population growth regulator” against *Aedes albopictus* under semi-field conditions. PLoS One 8(7):e67045. https://doi.org/10.1371/journal.pone.0067045 23843982 PMC3699564

[CIT0045] Paris V , Bell N, Schmidt TL, et al. 2023. Evaluation of In2Care mosquito stations for suppression of the Australian backyard mosquito, *Aedes notoscriptus* (Diptera: Culicidae). J. Med. Entomol. 60(5):1061–1072. https://doi.org/10.1093/jme/tjad099 37535973 PMC10496431

[CIT0046] Popko DA , Henke JA, Mullens BA, et al. 2018. Evaluation of two entomopathogenic fungi for control of *Culex quinquefasciatus* (Diptera: Culicidae) in underground storm drains in the Coachella Valley, California, United States. J. Med. Entomol. 55(3):654–665. https://doi.org/10.1093/jme/tjx233 29294059

[CIT0047] Richards SL , Balanay JAG, Fields M, et al. 2017. Baseline insecticide susceptibility screening against six active ingredients for *Culex* and *Aedes* (Diptera: Culicidae) mosquitoes in the United States. J. Med. Entomol. 54(3):682–695. https://doi.org/10.1093/jme/tjw231 28399272

[CIT0048] Sarkar M , Borkotoki A, Baruah I, et al. 2009. Molecular analysis of knock down resistance (*kdr*) mutation and distribution of *kdr* genotypes in a wild population of *Culex quinquefasciatus* from India. Trop. Med. Int. Health 14(9):1097–1104. https://doi.org/10.1111/j.1365-3156.2009.02323.x 19563477

[CIT0049] Savage HM , Smith GC, Moore CG, et al. 1993. Entomologic investigations of an epidemic of St. Louis encephalitis in Pine Bluff, Arkansas, 1991. Am. J. Trop. Med. Hyg. 49(1):38–45. https://doi.org/10.4269/ajtmh.1993.49.38 8352390

[CIT0050] Su T , Mullens P, Thieme J, et al. 2020. Deployment and fact analysis of the In2Care^®^ mosquito trap, a novel tool for controlling invasive *Aedes* species. J. Am. Mosq. Control Assoc. 36(3):167–174. https://doi.org/10.2987/20-6929.1 33600585

[CIT0051] World Health Organization (WHO). 2016. Test procedures for insecticide resistance monitoring in malaria vector mosquitoes. 2nd ed. Geneva, Switzerland: World Health Organization.

[CIT0052] Yee DA. 2008. Tires as habitats for mosquitoes: a review of studies within the eastern United States. J. Med. Entomol. 45(4):581–593. https://doi.org/10.1603/0022-2585(2008)45[581:tahfma]2.0.co;2 18714856

